# Detecting hypercoagulability in sepsis: thromboelastometry vs thrombomodulin-modified thrombin generation test

**DOI:** 10.1186/2197-425X-3-S1-A786

**Published:** 2015-10-01

**Authors:** VV Osovskikh, MS Vasileva, EV Kraisvetnaya, YA Namestnikov, OA Smirnova, AE Bautin

**Affiliations:** Research Center for Radiology and Surgical Technologies, Anesthesiology and Intensive Care, St.Petersburg, Russian Federation; North-West Medical Research Centre, Anesthesiology and Intensive Care, St.Petersburg, Russian Federation; Research Institute of Hematology and Transfusiology, Laboratory of Haemostasis, St.Petersburg, Russian Federation

## Introduction

Overt disseminated intravascular coagulation (DIC) develops in half of the septic patients by the fifth day, leading to double mortality in sepsis. Hypercoagulability, inevitably present before depletion of clotting factors and platelets, is hardly detected by available screening tests [1, 2]. This, in turn, prevents the development of targeted therapy at early stages of DIC. Global coagulation tests, such as the thromboelastography (TEG), thromboelastometry (TEM), thrombin generation (TG) are widely used to diagnose of a prothrombotic state. Thrombomodulin (TM)-modified TG also allows assessment of anticoagulant effect of the protein C (PC) pathway, but technical complexity limits it's use as a point of care (POC) test. At the same time, hypercoagulability in sepsis is often revealed by TEG or TEM, but the predictive value of these results remains uncertain [3].

## Objectives

The aim of the study is to compare the potential of TEM and TM+TG to detect hypercoagulability in sepsis.

## Methods

21 septic patients (12 men and 9 women, median age 59 years [IQR 46-64]) with TEM-diagnosed hypercoagulability were examined. TEM-criteria (ROTEM Delta) were: CTextem < 45, CTintem < 120, MCF> 72 mm, TPI (MCE/CFT) > 3,5. Platelet poor plasma was used to perform TG and TM+TG in duplicate (CAT, Thrombinoscope). Endogenous thrombin potential (ETP) and peak thrombin concentration (PT) were used to derive ETP-ratio and PT-ratio by dividing respective TG+TM value to TG value.

## Results

Thrombograms were obtained in 19 cases out of 21. Two samples failed probably due to contamination with heparin. Increased ETP and PT revealed only in one case. In other cases the generation of thrombin was either in the normal range or slightly reduced: median ETP 1530 nmol*min [IQR 1336 -1960], PT 244 nmol/l [IQR 204 - 293]. The degree of TM-modulated suppression of TG was abnormally low in 15 cases out of 19. Median ETP-ratio 0,86 [IQR 0,76-0,91], median PT-ratio 0,92 [IQR 0,88-0,97]. Thus, TEM- identified hypercoagulability was confirmed by both TG and TM+TG tests only in 16 cases out of 19 (84.2%). In our study only kinetic TEM-criteria (but not clot density criteria) were closely related to TG-criteria of hypercoagulability. For instance, clot formation time (CFT intem) highly correlated with ETP-ratio and PT-ratio (R^2^ = 0,82, p < 0.05). A trend towards shortening CFT intem in patients with early sepsis was reported earlier [2].

## Conclusion

Hypercoagulability during sepsis is often due to abnormality in PC pathway but not excessive thrombin generation. TEM appears to be sensitive POC tool to detect hypercoagulability in sepsis. TM-modified TG is a promising method to reveal underlying mechanism of coagulopathy in sepsis (and potentially to choose more targeted treatment), but requires further study.
